# Informed Consent in Veterinary Medicine: Ethical Implications for the Profession and the Animal ‘Patient’

**DOI:** 10.1007/s41055-017-0016-2

**Published:** 2017-08-21

**Authors:** Vanessa Ashall, Kate M. Millar, Pru Hobson-West

**Affiliations:** Centre for Applied Bioethics, School of Veterinary Medicine and Science and School of Biosciences, University of Nottingham, Nottingham, UK

**Keywords:** Veterinary ethics, Responsibility, Informed consent, Animal

## Abstract

Informed consent processes are a vital component of both human and veterinary medicine. Current practice encourages veterinarians to learn from insights in the human medical field about how best to achieve valid consent. However, drawing on published literature in veterinary and medical ethics, this paper identifies considerable differences between the *purposes* of veterinary and human medical consent. Crucially, it is argued that the legal status of animal patients as ‘property’ has implications for the ethical role of veterinary informed consent and the protection of the animal ‘patient’. It is suggested that veterinary informed consent should be viewed as an ethical pivot point where the multiple responsibilities of a veterinary professional converge. In practice, balancing these responsibilities creates considerable ethical challenges. As an example, the paper discusses the renewed call for UK veterinarians to make animal welfare their first priority; we predict that this imperative may increasingly cause veterinary informed consent to become an ethical pressure point due to tensions caused by the often conflicting interests of animals, owners and the veterinary profession. In conclusion, the paper argues that whilst gaining informed consent can often be presented as a robust ethical justification in human medicine, the same cannot be said in veterinary medicine. If the veterinary profession wish to prioritise animal welfare, there is an urgent need to re-evaluate the nature of authority gained through owner informed consent and to consider whether animal patients might need to be better protected outside the consent process in certain circumstances.

## Introduction

Informed consent has become an important part of both modern day veterinary practice and the practice of human medicine. Many countries, including the UK and the US require formalised consent procedures for veterinary treatment which appear similar to those required in the medical setting in a number of ways. Valid informed consent procedures in human medicine record a decision which is made voluntarily, with full knowledge and understanding of the relevant information (Delcarmen and Joffe 2005). Valid informed consent in veterinary practice requires similar considerations, for example the UK regulatory body, The Royal College of Veterinary Surgeons (RCVS), states within its Code of Professional Conduct:

*Informed consent, which is an essential part of any contract, can only be given by a client who has had the opportunity to consider a range of reasonable treatment options, with associated fee estimates, and had the significance and main risks explained to them* (RCVS 2014 *:Section 11.1)*

Here, veterinary professionals are being reminded that alongside the obtainment of a client’s signature on a form, there are certain criteria which must be fulfilled for legally valid consent to be given. Specifically, there is a need for full information provision, understanding and free choice during veterinary consent (Bateman 2007). With respect to the criteria which must be met for *valid consent* to be given, the veterinary and medical settings are identical.

Invalid consent is a concern for medical ethicists who have long argued that consenting parties may not be given adequate information or fully understand the proposed medical treatment (Byrne et al. 1988; Lavelle-Jones et al. 1993; Stanley et al. 1998). Since veterinary consent has identical validity criteria there is clear scope for veterinary professionals to learn from their medical colleagues when it comes to improving the *validity* of their consent procedures. Indeed, this is already occurring in the context of effective communication with studies of client-veterinarian interactions identifying poor communication skills being displayed by some veterinary professionals (Coe et al. 2008; Shaw et al. 2004b). Several authors have explicitly argued for veterinarians to improve communication in line with their human medical colleagues (Shaw et al. 2004a; Bonvici and Keller 2006).

The *legal* benefits of gaining valid consent are also shared by both professions. Medical informed consent procedures play a vital role in legal protection for clinicians from patient accusations of assault or malpractice (Jackson 2010). Veterinary informed consent is also used to provide legal protection for both the owner and the vet (Flemming and Scott 2004) and for some, veterinary informed consent has been primarily defined as a risk management tool (Martin 2006). The UK RCVS has identified improved veterinary informed consent practices as an important route to reducing official complaints from animal owners (RCVS 2008).

In summary, there is plenty of scope for fruitful comparison between informed consent processes in the veterinary and medical settings, in particular with regards to the criteria which ensure the validity of informed consent and the role that a robust consent procedure can play in avoiding legal proceedings. However, beyond validity criteria and litigation, there remain key differences in the *purpose* of informed consent in the medical and veterinary settings and these differences have significant ethical implications, particularly for the way in which animal patients are protected. The aim of this paper is to re-evaluate the purpose of consent in a veterinary setting and to make important differences clear, sounding a note of caution about the extent to which consent in human and animal medicine contexts can be compared and translated.

## What is the Purpose of Informed Consent in Medical Practice?

Reflecting on human medical practice, consent is the mechanism though which human beings exercise their rights of determination over their own bodies. The relationship between bodily rights and consent in the medical context were addressed by Judge Benjamin Cardozo in the case of Mary Schloendorff v the Society of New York Hospital in 1914:

*Every human being of adult years and sound mind has a right to determine what shall be done with his own body; and a surgeon who performs an operation without his patient’s consent commits an assault.* (Schloendorff v Society of New York Hospital, 211 NY 125 [NY 1914])

The widespread adoption of formal informed consent processes is generally attributed to the outcomes of the Nuremberg trials, which addressed infamous atrocities suffered by the subjects of medical research conducted by the Nazi regime (Murphy and Dingwall 2007; Rogers et al. 2012). Public outcry and outrage within the medical profession led to the adoption of the Nuremberg Code (1947), establishing informed consent as a guiding ethical consideration in medical research. Informed *patient* consent was later developed to protect autonomous choice in medical patients (Beauchamp and Childress 2013).

Autonomy is an important ethical concept and translates as *‘self-rule’*. In their influential book *Principles of Biomedical Ethics*, Beauchamp and Childress propose that in order to be an autonomous agent (i.e. someone able to consent) one must be able to act:

1. *Intentionally*
2. *With understanding, and*
3. *Without controlling influences that determine their action*

(Beauchamp and Childress 2013; p.104).

Autonomous individuals are suggested to be acting with sufficient information, understanding and of their own free will. The similarity between these criteria for autonomy and those earlier identified for valid informed consent illustrates how informed consent processes aim to protect autonomous choice.

In practice, how patients’ choices should interact with the needs of others and the medical profession can be ethically complex. Whilst the work of Beauchamp and Childress has inevitably been subjected to academic scrutiny (for example see Lee 2010; Karlsen and Solbakk 2011), for reasons of simplicity, only this view of how autonomous choice (and thus consent) should influence medical decision making is discussed here. Continuing a principled approach, respect for autonomy is viewed as just one of four guiding ethical principles alongside beneficence (doing good), non-maleficence (preventing harm) and justice (fairness) (Beauchamp and Childress 2013). These four principles must be balanced during the ethical justification of an action, meaning respect for patient autonomy may be used to counter arguments that result from the application of one of the other principles, such as enhancing the wellbeing (doing good) of a population through harming an individual in medical research. On the other hand, if a patient provides valid informed consent this may offset concerns raised through the application of other ethical principles such as non-maleficence. Patient informed consent, therefore, can have a particularly important or even pivotal role in the weighing and justification of potentially harmful procedures such as the donation of healthy tissues and organs or participation in medical research.

In summary, medical informed consent procedures aim to protect an individual’s rights to make autonomous choices concerning their own body. As such, medical consent might be shown to protect a patient from procedures they do not want or to allow choice over the degree of risk they are willing to accept in medical or research interventions. With this medical context considered, how comparable is the purpose of informed consent in the veterinary setting?

## What is the Purpose of Informed Consent in Veterinary Practice?

Whilst veterinary consent practices have attracted some attention in the literature, with a small number of articles addressing the legal requirements and implications of veterinary consent, there has been very little discussion of the ethical nature and purpose of veterinary consent (Fettman and Rollin 2002). For example Flemming and Scott (2004) suggest that:

*The purpose of informed consent is to provide the client enough information that he or she can make an informed decision for or against the recommended health care*.

(Flemming and Scott 2004; p.1439)

Flemming and Scott describe how informed consent processes improve an owner’s decision making, but what is the purpose of requiring veterinary informed consent? Who is protected and why? We have described how medical informed consent protects the human patient’s rights to bodily integrity and that treatment without consent could result in accusations and possible charges of assault being brought. In the veterinary context however:

*Any unconsented harm caused by a veterinarian to an animal owned by another individual would likely violate a property right and therefore be a form of civil wrong. It is on this basis that the informed consent doctrine applies to animals and, thereby, to veterinarians.* (Flemming and Scott 2004; p. 1436 )

Flemming and Scott (2004) thus clearly illustrate how the purpose of the veterinary consent process differs from medical informed consent. Veterinary consent protects a right over property enjoyed by the animal’s owner, however, it does not protect any legal or moral rights enjoyed by the animal ‘patient’ themselves. Unlike the assault charges faced by doctors acting without consent, a veterinarian acting without proper consent would be accused of damaging (animal) property.

Historically, this focus on property rights makes sense if one remembers that veterinary consent was traditionally associated with making choices which preserved the economic value of a farmer’s stock (Fettman and Rollin 2002). Veterinary informed consent still has the same legal function today with animals being considered (for most legal purposes) as *special* property (Flemming and Scott 2004b) However, in more recent years the relationship between humans and animals in the veterinary setting has altered significantly, with more focus on animals as companions and a broader understanding of the animal’s value, such as an emotional significance, rather than only economic value (Hueston 2016; Hobson-West and Timmons 2016). Indeed, Fettman and Rollin specifically link this shift to changes in the ‘paradigm’ of consent, suggesting that:

*The growing populations of companion animals and the shift in veterinary-client relationships to one founded in the human-animal bond has effectively altered the paradigm of informed consent, even if the profession has not yet fully acclimated to this important change. In this instance, the economic foundation of informed consent has largely been replaced by an emotional and moral one, wherein risk and benefit are judged in terms of quality of life, empathy, anthropomorphism, and considerations for informed consent not unlike those for parent, child, and paediatrician.*

(Fettman and Rollin 2002; p.1386)

If we accept Fettman and Rollins’ argument then we accept that the purpose of informed consent is influenced by the nature of the relationship between the owner and the animal. However, what remains constant regardless of the type of owner-animal relationship is that veterinary informed consent is designed to allow the owner to make autonomous choices which protect the value of their animal (whether economic, emotional, etc.) *to themselves*. We will later explore the relevance of Rollin’s comparison between vets and paediatricians when exploring the implications of informed consent for veterinary professionals.

## Veterinary Informed Consent: Implications for Animal ‘Patients’

If a medical patient is protected by a rigorous informed consent process, what does consent imply for protection of a veterinary ‘patient’? Passantino et al. (2011) observes that the animal patient cannot make their own medical decisions and suggests that veterinary consent ‘*is not based on the principle of individual autonomy, since* [autonomy] *expresses the subject’s self-determination, which the animal species is not endowed with’* (Passantino et al. 2011; p.129). As argued above, it is claimed here that veterinary informed consent *does* aim to protect individual autonomy, but that of the animals’ owner, rather than the animal themselves. Passatino recapitulates that informed consent does not protect *patient autonomy* in the veterinary setting, because of the lack of capacity of animal patients to make their own medical decisions. This is a central point and again highlights the difference in nature of consent practices; the autonomous decision made in the veterinary context is not made by the ‘patient’ but by their owner. Importantly, when considering the implications for the animal patient, a superficially comparable situation does exist in human medicine where children or the mentally incapacitated are treated by medical professionals with the consent of a parent or guardian.

Medical ethicists have paid a great deal of attention to how well bodily rights are protected for humans who cannot consent to their medical treatment. The relatives or guardians of such individuals have legal rights to make decisions on their behalf, however, it is well recognised that a family member might not always promote the best interests of the patient when making third party medical decisions (Downie and Randall 1997). Crucially, doctors cannot tailor medical interventions according to the preferences of any consenting party (Birchley 2016), rather they are obliged to act in the best interests of their patient only and third party consent cannot be used against the patient’s interests. If a medical intervention which is argued to be in the patient’s best interests carries a significant risk of harm, an independent assessment (for example conducted by a court of law) may be required before doctors can proceed (WHO 2010), even when third party consent has been provided.

As in the human medical setting, it is of course a common scenario that an animal owner and veterinary surgeon will both want what is best for the animal patient. However, it is important to accept that the autonomous decision of an animal owner made according to their legal rights might also be influenced by other factors. As highlighted above, an owner is giving informed consent to veterinary treatment in order to protect the value of their animal as perceived by themselves. This will not necessarily always coincide with what is in the animal’s best interests. Even where animals are primarily considered to be of emotional value, as in the case of companion animals, a strong emotional bond might result in requests for prolonged palliative treatment or surgical procedures which, it has been argued, might not always reflect what is actually best for the animal patient (Palmer et al. 2012). It is precisely this concern which underpins the need for an independent judgement in some cases which involve human patients who are not able to provide consent for themselves, but which is not currently required in the veterinary setting.

In summary, there are considerable implications for the wellbeing of animal ‘patients’ compared to human patients when considering the function of veterinary consent processes. The difference arises from the fact that animals are not autonomous and cannot consent for themselves and that the third party consent provided by their owner aims to protect their interest or perception of the value of their animal. Whilst non autonomous humans still possess rights over their own body which cannot be overruled by third party consent, the same situation is not true for animals, and some animal owners may request veterinary interventions which are not necessarily in their animal’s best interests. The veterinary informed consent process aims to protect the owner’s right to make such choices, not to protect the animal patient. It is this inherent tension which explains why veterinary informed consent should be understood as an *ethical pivot point*. The article now moves to consider the implications of this argument for the veterinary profession.

## Veterinary Consent: Implications for the Veterinary Profession

We have highlighted the obligations of the medical profession to protect the best interests of human patients who cannot consent to treatment themselves and how this obligation limits the power of third party consent in human medicine. How comparable are the obligations of veterinary professionals and what implications do they have for the scope of authority of consent given by animal owners? Given the international variation which exists in veterinary professional legislation, the UK veterinary profession is examined here as one example. We hope that this paper will encourage more comparative work and reflection on the situation beyond the UK.

The UK veterinary profession has long maintained that animal welfare is its primary concern. The oath which must be taken by new veterinary surgeons in the UK states that:

.. *ABOVE ALL, my constant endeavour will be to ensure the health and welfare of animals committed to my care. *(RCVS 2014)

But the code of conduct further recognises that veterinary surgeons’ professional responsibilities to animals and their owners may, at times, conflict with each other and create ethical dilemmas. The veterinary surgeon is therefore advised to balance their professional responsibilities, having a first regard to animal welfare. The likelihood and accuracy of the good intentions of such a code is not universally accepted. Hewson (2006) commented that ‘*It is impossible to know whether taking an oath makes a difference to an individual veterinarian’s attention to animal welfare’* (Hewson 2006; p.809). More radically, it has even been suggested that this declaration ‘*is at best delusional since its principle claim – to champion the welfare of animals –cannot be substantiated*’ (Coffey 2008; p.266). Coffey (2008) argues that by condoning and supporting the domestication of animals the profession becomes a tool of animal oppression; complicit by default in the abuse of animals. Others have taken a less polarised view but still argue that there are clearly likely to be conflicting motivations experienced by veterinary surgeons that aim to advocate for animals and yet must also make a profit from their services (Main 2006). These perspectives again articulate the tensions which make the process of veterinary informed consent an ethical pivot point. In short, if the profession do abide by their oath and prioritise animal welfare, what does this mean for veterinary informed consent?

This question is indeed a very relevant one in the UK, where the role of the profession as animal welfare champions has recently been revisited by the British Veterinary Association (BVA) through the publication of its ‘Vet Futures’ project (BVA 2016). The BVA hopes the project will help shape the profession and provide a clear direction for development in the coming years. The report outlines numerous recommendations and those at the top of the list are:

1. Develop and promote an animal welfare strategy for the veterinary Profession.
2. Enhance moral reasoning and ethical decision-making in education, policy-making, practice-based research and everyday veterinary work
  (BVA 2016; p6).

Such a renewed focus on ethics is welcomed, and some may feel this is long overdue, for a profession which faces a myriad of ethical conflicts (Morgan 2007). In the report the BVA’s pledge is to “*support members to maximise their animal advocacy potential and achieve good welfare outcomes for animals”* (p.3). However, Fettman and Rollin (2002) argue that the animal advocate (or paediatrician model) is but one of many ethical roles a veterinary surgeon plays. Considering the BVA’s interpretation then, we pause to consider what a renewed focus on animal welfare might mean for veterinary informed consent.

Authors have previously argued that veterinary surgeons, as animal welfare advocates, would have an obligation to positively influence owner decision-making by promoting the course of action which is best for the animal (Main 2006). Fettman and Rollin (2002) go further and ask us to consider the implications of fully following through on patient advocacy. In short, there may be no need for veterinary informed consent at all:

*An extreme view of patient advocacy might lead one to conclude that there is no need for informed consent from the owner, based on the assumption that the veterinarian knows what is best and need not explain his/her decision-making process in selecting treatment. In fact, there are situations where veterinarians are asked to serve only as a patient advocate, when an owner adamantly insists that anything be done for his/her beloved pet, regardless of the cost or inconvenience to the owner, as long as the animal does not suffer unnecessarily or without clear benefit*. (Fettman and Rollin 2002; p.1388)

Indeed, in emergency situations, veterinary surgeons in the UK (and other countries, including Denmark) are obliged to act in the animal’s interests, both without payment and without the owner’s consent (Sandøe et al. 2016). However, if animal advocacy is to be promoted as the primary role of the vet then veterinary informed consent will have a reduced authority overall and cannot be presented as the sole ethical justification for *any* veterinary procedures, unlike comparable medical procedures, because its purpose is not to protect animals. Passantino and colleagues (2011) make a similar claim suggesting that:

*Choice of the treatment lies with the Veterinarian, because, for his/her professional skills, technical and scientific knowledge of medicine, he/she stands on a higher level than the owner of the animal. Once the treatment has been chosen, the veterinarian must propose it to the client, who might not accept it. In this case, the veterinarian is free of any professional obligation and is not obliged to carry out any treatments that the client may consider suitable, but which are against the physician’s “science and conscience”* (Passantino et al. 2011; p. 130)

These arguments place the veterinarian in a comparable position to the paediatrician, who is motivated solely by the best interests of his non-autonomous patient (Fettman and Rollin 2002) and this ‘paediatrician’ comparison has indeed been made directly by the BVA in support of its animal welfare strategy:

*A comparison with paediatricians is relevant: we do not expect a doctor to approach questions of a young child’s health and welfare with a parent’s wishes, or the doctor’s career development, as the focus of decision-making. We expect a paediatrician to prioritise the best interests of their young patients, enabled by the child’s parents/guardians and the doctor’s skills and resource. Promoting a patient’s best interests sometimes requires ethically appropriate influencing of animal owners. (BVA* 2016 *: p.20)*

This vet-paediatrician comparison cannot hold true unless the ethical value of an owner’s consent is limited to that of third party consent given in human medicine. According to the ethical principles upheld, the owner could therefore not overrule the veterinarian’s obligation to provide whatever is best for the animal. The veterinary profession would have to accept that, as in human medicine, third party consent is *not* the ethical justification for veterinary procedures, which are conducted solely on the basis that they are in the animal’s best interests. The question which then arises is what are the implications of this situation for the profession and is such an approach feasible, given the current status and role of practising veterinarians?

The BVA argument is potentially radical in resetting the traditional focus on the legal rights of the owner who, by law, is still entitled to make decisions which protect the value of their property as they view it (e.g. economic, emotional, etc). Indeed, prioritising the best interests of the animal might actually cause negative economic value (a financial harm), and place a significant financial burden on the animal’s owners. In its current form, veterinary informed consent undoubtedly has *legal* significance which would prevent the vet from acting against the wishes of the owner, assuming that the owner is not infringing other animal welfare legislation (e.g. the UK Animal Welfare Act 2006). Furthermore, there will be examples where it is not at all clear what is in the animal’s best interests. Practical examples of such a conflict are not hard to come by, as Rollin powerfully summarises:

*In a given day, a morally aware veterinarian may face one client who wants a perfectly healthy dog killed and another who will not consider euthanasia of a terminally ill animal in pain* (Rollin 2002; p.1146)

In these examples, we can imagine the further tension felt by the veterinary surgeon where the current emphasis placed on trying to promote an animal’s best interests would mean potentially acting against an owner’s wishes. This means that the informed consent process is further complicated, becoming an *ethical pressure point*, where factual uncertainty, the ethical obligations of the vet and the owner’s legal rights to make choices collide. It must be accepted that there is a practical difference between these two scenarios in that a veterinary surgeon may conceivably refuse to enthanase a healthy animal but is less likely to euthanase a sick animal against the owner’s wishes. However, even before the new UK emphasis on animal advocacy, Main (2006) highlighted the significant lack of support UK veterinary surgeons have when grappling with either of these ethical dilemmas. In response to the RCVS code of professional conduct Main states:

*The veterinary surgeon is not directed to refuse completely an inappropriate request for euthanasia or to euthanase an animal against the owner’s consent. Rather, the veterinary surgeon is advised to refer to other colleagues in the first case or to contact the authorities in the second case…. in most circumstances, a veterinary surgeon on his or her own has very limited powers without an owner’s consent’.*

(Main 2006; p.63)

This analysis points to informed consent as a growing ethical pressure point. Not only might vets feel unsupported in such clinical situations, but professional respect for the current articulation of the veterinary informed consent process might even be experienced as *disempowering* to a veterinary surgeon who is now compelled to advocate for animals. The nature, purpose and operationalisation in practice of veterinary informed consent therefore need urgent re-evaluation.

## Conclusions

There are clear similarities in the processes of gaining valid informed consent which protects autonomous decision-making in both human and veterinary professional settings. However, the purpose of informed consent and therefore its ethical significance is notably different in the medical and veterinary contexts. Whilst medical consent protects a patient’s rights to make autonomous decisions concerning their own body, veterinary informed consent aims to protect an owner’s right to make autonomous decisions concerning their legal property. We have identified the implications of the different meaning of informed consent for the protection of ‘patients’ in the veterinary setting.

The consent procedure in the veterinary setting is here described as an ethical pivot point, where multiple competing interests must be weighed to determine a defensible course of action. Consequently we have argued that recent emphasis in the UK on a veterinary surgeon’s role as animal advocate needs to be considered in light of the complex ethical and legal obligations that veterinary surgeons hold, and which are played out during the veterinary consent process. As we have shown, promoting veterinarians as animal advocates has a considerable effect on the meaning and value of an owner’s informed consent. This potential shift in a veterinarian’s role may necessitate considerable change to veterinary practice and veterinary legislation and furthermore appears to be inconsistent with the current legal status of animals.

In the meantime, what is urgently needed is a thorough re-evaluation of the scope of authority of veterinary informed consent. It appears that whilst veterinary surgeons might find it hard to challenge an owner’s decisions, a full appreciation of the purpose of veterinary informed consent reveals that owner consent cannot be considered an ethically justifiable reason to concede to certain requests. There is little current work which explores the decision making rationale of veterinary surgeons in this specific situation and this could be an important area for future empirical research. Indeed, sociological research in the form of ethnographic (Morris, 2012), interview (Ashall and Hobson-West 2017) and survey work (Kondrup et al. 2016) is beginning to open up discussion on the societal complexities of veterinary decision making in a variety of ethically charged scenarios, including euthanasia, donation and non-payment. Further empirical work would significantly inform the argument made in this paper: In short, that a lack of appreciation of the differences in meaning of informed consent given for sometimes identical medical and veterinary practices may result in a misunderstanding of the relevance of veterinary informed consent to ethical decision making, both by veterinary professionals and animal owners.

Finally, even in (human) medical ethics, where patient consent is valued highly, there is a very clear understanding that informed consent should *not* be considered the sole ethical concern (Manson and O'neill 2007; Dawson 2004). That informed consent might even be irrelevant as an ethical justification in certain circumstances is promoted by medical ethicists; Beauchamp and Childress, in their most recent edition (2013), “*do not presume that consent is either necessary or sufficient for certain interventions to be justified…*” (2013, p. 110). Critics of medical consent practices argue against the absolute prioritisation of respect for autonomy above other ethical principles, such as beneficence, non-maleficence and justice (Corrigan 2003). Arguably, in the veterinary world, where the patient is not the one consenting, this argument could be seen as *even more* powerful and pivotal. For the veterinary profession, this means that the community should be placing at least equal emphasis on preventing harm and the fair treatment of animals as that placed on allowing owners to make autonomous choices. We therefore ask the veterinary profession to reflect on conclusions drawn by ethicists in the medical field who argue that:

*Informed consent plays a critical role in clinical medicine but that other models of decision making deserve consideration under particular circumstances* (Delcarmen and Joffe 2005: pp 636)

In human medicine, an independent body such as a court has authority in cases where the ethics of treatment with only third party consent is unclear. These types of mechanisms may have an important role to play in the veterinary field. In fact there have been recent calls for an ‘animal ombudsman’ (Sweeney 2015) and the RCVS are currently trialling the use of an independent ethical review panel to deliberate on questions raised by veterinary clinical research (RCVS, 2016). Such concepts might well be usefully applied to the ethical questions which are commonly encountered by veterinary professionals. There are currently limited ‘safe spaces’ (Millar and Hobson-West 2015) where veterinarians can discuss and develop their ethical viewpoints. This needs to be addressed with the aim of debating justifiable ethical stances at the professional, practice and individual level (Mullan and Main 2001; Millar 2011).

Going forward, many organisations and individuals need to consider ways to support the modern veterinary professional and equip them to develop their ethical reasoning skills. Veterinarians should be supported to make robust ethical decisions which encompass a deeper understanding of the ethical principles underlying their professional obligations.

## References

[CR1] Ashall, V. & Hobson-West, P. 2017. Doing good by proxy: Human-animal kinship and the ‘donation’ of canine blood. *Sociology of Health and Illness* 39 (6): 908–922. 10.1111/1467-9566.12534 PMC5516241 28164318

[CR2] Bateman SW (2007). Communication in the veterinary emergency setting. Veterinary Clinics: Small Animal.

[CR3] Beauchamp TL, Childress JF (2013). Principles of biomedical ethics.

[CR4] Birchley G (2016). Harm is all you need? Best interests and disputes about parental decision-making. Journal of Medical Ethics.

[CR5] Bonvici K, Keller VF (2006). Academic faculty development: The art and practice of effective communication in veterinary medicine. JVME.

[CR6] BVA. 2016. Vets speaking up for animal welfare: British Veterinary Association animal welfare strategy. Available at https://www.bva.co.uk/uploadedFiles/Content/News,_campaigns_and_policies/Policies/Ethics_and_welfare/BVAanimal-welfare-strategy-feb-2016.pdf. Accessed 15 Aug 2017.

[CR7] Byrne DJ, Napier A, Cuscheri A (1988). How informed is signed consent?. British Medical Journal.

[CR8] Coe JB, Adams CL, Bonnett BN (2008). A focus group study of veterinarians’ and pet owners’ perceptions of veterinarian-client communication in companion animal practice. JAVMA.

[CR9] Coffey D (2008). The veterinary profession. Journal of the Royal Society of Medicine.

[CR10] Corrigan O (2003). Empty ethics: The problem with informed consent. Sociology of Helath and Illness.

[CR11] Dawson AJ (2004). Methodological reasons for not gaining prior informed consent are sometimes justified. BMJ.

[CR12] Delcarmen MG, Joffe S (2005). Informed consent for medical treatment and research: A review. The Oncologist.

[CR13] Downie RS, Randall F (1997). Parenting and the best interests of minors. The Journal of Medicine and Philosophy.

[CR14] Fettman MJ, Rollin BE (2002). Modern elements of informed consent for general veterinary practitioners. JAVMA.

[CR15] Flemming D, Scott JF (2004). The informed consent doctrine: What veterinarians should tell their clients. JAVMA.

[CR16] Hewson CJ (2006). Veterinarians who swear: Animal welfare and the veterinary oath. Candian Veterinary Journal.

[CR17] Hobson-West, P., and S. Timmons. 2016. Animals and anomalies: An analysis of the UK veterinary profession and the relative lack of state reform. *The Sociological Review* 64 (1): 47–63.

[CR18] Hueston W (2016). Veterinary medicine: Public good, private good or both?. Veterinary Record.

[CR19] Jackson E (2010). Medical Law.

[CR20] Karlsen JR, Solbakk JH (2011). A waste of time: The problem of common morality in principles of biomedical ethics. Journal of Medical Ethics.

[CR21] Kondrup SV, Anhøj KP, Rødsgaard-Rosenbeck C, Lund TB, Nissen MH, Sandøe P (2016). Veterinarian's dilemma: A study of how Danish small animal practitioners handle financially limited clients. The Veterinary Record.

[CR22] Lavelle-Jones C, Byrne DJ, Rice P, Cuschieri A (1993). Factors affecting quality of informed consent. BMJ.

[CR23] Lee MJ (2010). The problem of 'thick in status, thin in content' in Beauchamp and Childress' principlism. Journal of Medical Ethics.

[CR24] Main D (2006). Offering the best to patients: Ethical issues associated with the provision of veterinary services. The Veterinary Record.

[CR25] Manson NC, O'neill O (2007). Rethinking informed consent in bioethics.

[CR26] Martin EA (2006). Managing client communication for effective practice: What skills should veterinary graduates have acquired for success?. JVME.

[CR27] Millar KM, Wathes CM, Corr SA, May SA, McCulloch SP, Whiting MC (2011). Ethics and ethical analysis in veterinary science and medicine: The development and application of the ethical matrix method. Veterinary and animal ethics: Proceedings of the first international conference on veterinary and animal ethics.

[CR28] Millar, K.M., and P. Hobson-West. 2015. Supporting veterinarians to engage with ethics in practice: Safe spaces and the role of the veterinary ethicist. Proceedings of the international conference VETHICS for veterinary ethics: Tierschulz und Tiermedizin. Animal welfare and veterinary medicine. 17-18 September 2015, Wien/Vienn.

[CR29] Morgan CA (2007). Ethical dilemmas in veterinary medicine. Veterinary Clinics: Small Animal.

[CR30] Morris P (2012). Managing pet Owners' guilt and grief in veterinary euthanasia encounters. Journal of Contemporary Ethnography.

[CR31] Mullan S, Main D (2001). Principles of ethical decision-making in veterinary practice. Practice.

[CR32] Murphy E, Dingwall R (2007). Informed consent, anticipatory regulation and ethnographic practice. Social Science & Medicine.

[CR33] Palmer C, Corr S, Sandøe P (2012). Inconvenient desires: Should we routinely neuter companion animals?. Anthrozoös.

[CR34] Passantino A, Quartarone V, Russo M (2011). Informed consent in veterinary medicine: Legal and medical perspectives in Italy. Open Journal of Animal Sciences.

[CR35] RCVS. 2008. Don’t become a complaints statistic. *RCVS News: A special report of the Royal College of Veterinary Surgeons*. Available at https://www.rcvs.org.uk/publications/rcvs-news-extra.../rcvsnewsxtra_feb08.pdf. Accessed on 15.8.17

[CR36] RCVS. 2014. *RCVS Code of Professional Conduct for Veterinary Surgeons.* http://www.rcvs.org.uk/advice-and-guidance/code-of-professional-conduct-for-veterinary-surgeons/supporting-guidance/miscellaneous/. Accessed 1 Oct 2014.

[CR37] RCVS. 2016. Ethics review panel. https://www.rcvs.org.uk/about-us/committees/ethics-review-panel/. Accessed 14 Apr 16.

[CR38] Rogers W, Mackenzie C, Dodds S (2012). Why bioethics needs a concept of vulnerability. International Journal of Feminist Approaches to Bioethics.

[CR39] Rollin BE (2002). The use and abuse of Aesculapian authority in veterinary medicine. JAVMA.

[CR40] Sandøe P, Corr S, Palmer C (2016). Companion animal ethics.

[CR41] Shaw JR, Adams CL, Bonnett BN (2004). What can veterinarians learn from studies of physician-patient communication about veterinarian-client-patient communication?. JAVMA.

[CR42] Shaw JR, Adams CL, Bonnett BN, Larson S, Roter DL (2004). Use of the Roter interaction analysis system to analyze veterinarian-client-patient communication in companion animal practice. JAVMA.

[CR43] Stanley BM, Walterasn DJ, Maddern GJ (1998). Informed consent: How much information is enough? *Ausr. N.Z*. J. Surg..

[CR44] Sweeney, N. 2015. *Why we need an animals’ ombudswoman. From AWSEL to Practice-Maximising the impact*. Presentation to AWSELVA Conference: Bristol.

[CR45] WHO. 2010. WHO guiding principles on human cell, tissue and organ transplantation. World Health Organisation. Available at http://www.who.int/transplantation/Guiding_PrinciplesTransplantation_WHA63.22en.pdf?ua=1. Accessed on 15.8.17 10.1097/TP.0b013e3181ec29f0 20664493

